# CRISPR-Cas12a-Assisted Recombineering in Bacteria

**DOI:** 10.1128/AEM.00947-17

**Published:** 2017-08-17

**Authors:** Mei-Yi Yan, Hai-Qin Yan, Gai-Xian Ren, Ju-Ping Zhao, Xiao-Peng Guo, Yi-Cheng Sun

**Affiliations:** a MOH Key Laboratory of Systems Biology of Pathogens, Institute of Pathogen Biology, and Center for Tuberculosis Research, Chinese Academy of Medical Sciences and Peking Union Medical College, Beijing, China; b Department of Histology and Embryology, Bengbu Medical College, Bengbu, Anhui, China; Goethe University Frankfurt am Main

**Keywords:** Cas12a, recombineering, Mycobacterium smegmatis, Yersinia pestis

## Abstract

Clustered regularly interspaced short palindromic repeat (CRISPR)-Cas12a (Cpf1) has emerged as an effective genome editing tool in many organisms. Here, we developed and optimized a CRISPR-Cas12a-assisted recombineering system to facilitate genetic manipulation in bacteria. Using this system, point mutations, deletions, insertions, and gene replacements can be easily generated on the chromosome or native plasmids in Escherichia coli, Yersinia pestis, and Mycobacterium smegmatis. Because CRISPR-Cas12a-assisted recombineering does not require introduction of an antibiotic resistance gene into the chromosome to select for recombinants, it is an efficient approach for generating markerless and scarless mutations in bacteria.

**IMPORTANCE** The CRISPR-Cas9 system has been widely used to facilitate genome editing in many bacteria. CRISPR-Cas12a (Cpf1), a new type of CRISPR-Cas system, allows efficient genome editing in bacteria when combined with recombineering. Cas12a and Cas9 recognize different target sites, which allows for more precise selection of the cleavage target and introduction of the desired mutation. In addition, CRISPR-Cas12a-assisted recombineering can be used for genetic manipulation of plasmids and plasmid curing. Finally, Cas12a-assisted recombineering in the generation of point mutations, deletions, insertions, and replacements in bacteria has been systematically analyzed. Taken together, our findings will guide efficient Cas12a-mediated genome editing in bacteria.

## INTRODUCTION

The clustered regularly interspaced short palindromic repeat and CRISPR-associated protein (CRISPR-Cas) system is a prokaryotic adaptive immune system that confers resistance to foreign genetic elements (1–3). Class 1 CRISPR systems (comprising types I, III, and IV) typically form multisubunit protein-CRISPR RNA (pcrRNA) complexes, whereas class 2 systems (comprising types II, V, and VI) use a single crRNA-guided protein for target interference (2). Recently, engineered Cas nucleases, including CRISPR-Cas9 and CRISPR-Cas12a (Cpf1), have been widely adopted as versatile genome editing tools in many organisms (4–6). The CRISPR-Cas9 system, categorized as class 2 type II, consists of a Cas nuclease (Cas9), a *trans*-activating CRISPR RNA (tracrRNA), and a crRNA (4, 5, 7). The CRISPR-Cas9 system generates Cas9-mediated double-strand cleavage of the target sequence guided by the crRNA and tracrRNA. Cas12a, which is a type V-A endonuclease of the class 2 CRISPR-Cas system, is a dual nuclease that is involved in crRNA processing, target-site recognition, and DNA cleavage (4, 8). Target DNA binding in most DNA-targeting CRISPR-Cas systems is dependent on the initial recognition of a protospacer adjacent motif (PAM), a short (3 to 5 bp) DNA sequence adjacent to the protospacer (target) site that is complementary to the crRNA spacer segment (9). Cas12a recognizes thymidine-rich PAM sequences (YTN) and can be guided by a single crRNA without an additional tracrRNA (4, 8, 10). It has been recently used for genome editing in mammals, plants, and bacteria (11–15).

Recombineering, a method based on genetic engineering through homologous recombination, has provided new ways to manipulate bacterial genomes (16–18). Although the method is effective, antibiotic resistance genes must be used to isolate recombinants. To generate markerless mutations, antibiotic resistance genes must be cured by a technique involving a site-specific recombinase or resolvase (17, 19–21). Typically, these manipulations require multiple steps and create a chromosomal “scar” (e.g., at the LoxP recognition site). Thus, coupling a CRISPR-Cas system with recombineering represents a simple and highly efficient genome editing method in bacteria (6, 22–24). For example, the CRISPR-Cas9 system has been used to assist recombineering in Escherichia coli and Lactobacillus reuteri (25–27). By coupling the CRISPR-Cas9 system with lambda Red recombineering, highly efficient recombination can be achieved without an antibiotic marker using single-stranded DNA (ssDNA) or double-stranded DNA (dsDNA) generated by PCR (25–27). However, Cas9 typically uses a G-rich PAM sequence, such as NGG, which hinders the precise application of CRISPR-Cas9-assisted engineering of point mutations using ssDNA.

Mycobacterium tuberculosis is the causative agent of tuberculosis (TB), which causes 1.5 million deaths worldwide every year. Genetic manipulation of M. tuberculosis is limited by the bacterium's low growth rate, inefficient DNA uptake, and high frequency of illegitimate recombination. Recently, recombineering was successfully applied to the genetic manipulation of mycobacteria (18). Expression of the recombination proteins gp60 and gp61 from the mycobacteriophage Che9c increases the efficiency of recombination and facilitates allelic exchange in both Mycobacterium smegmatis and M. tuberculosis (18). dCas9, an endonuclease-deficient Cas9 that contains two mutations (D10A and H840A) in the nuclease domains, has been used to regulate gene expression in mycobacteria (28–30). However, CRISPR-Cas9-assisted genome editing has not yet been applied to this group of microorganisms.

Thus, we explored whether the CRISPR-Cas12a system could be used to assist recombineering in bacteria, particularly in mycobacteria. Our results show that CRISPR-Cas12a-assisted recombineering can rapidly and efficiently generate point mutations, deletions, and insertions in Escherichia coli, Yersinia pestis, and Mycobacterium smegmatis. The technique simplifies the construction of markerless mutations in bacteria, avoids the creation of chromosomal scars and is especially effective for the sequential recombineering of multiple genes in bacteria.

## RESULTS

### CRISPR-Cas12a-assisted recombineering in E. coli.

**FIG 1:**
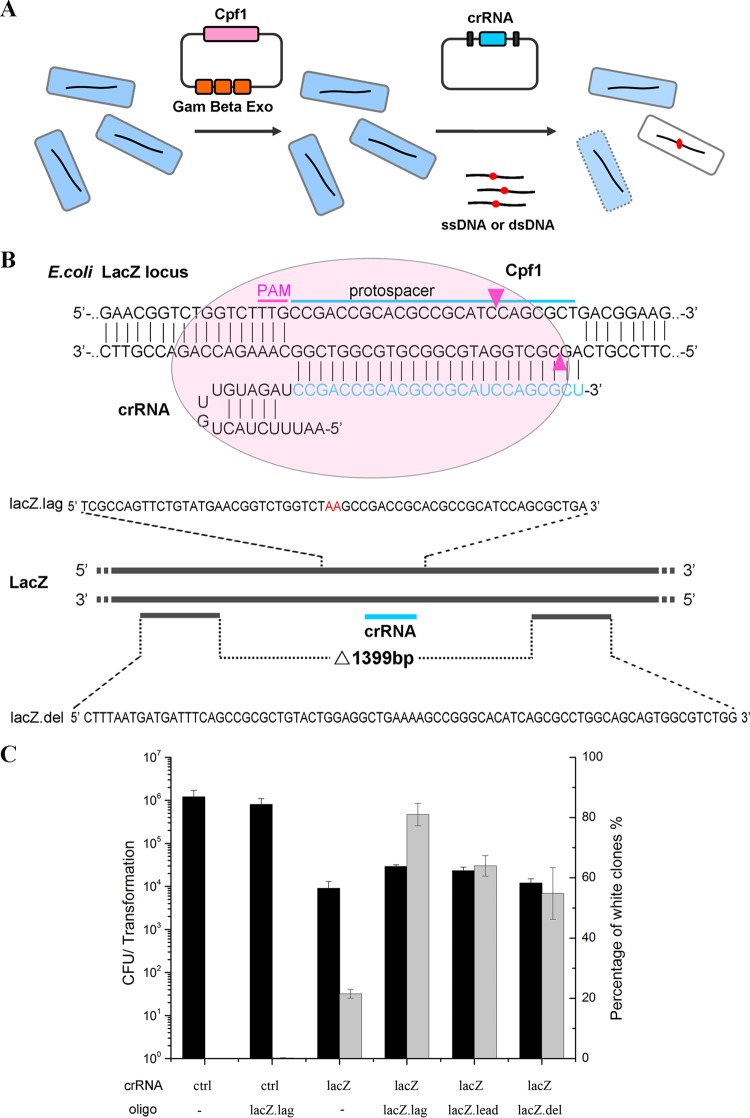
CRISPR-Cas12a-assisted genome editing in E. coli. (A) Schematic of CRISPR-Cas12a coupled with the λ Red system in E. coli. First, Cas12a and recombinase were expressed in bacteria. Then, the crRNA-expressing plasmid and ssDNA (or dsDNA) were transformed into the cell. When the crRNA targets the E.coli *lacZ* locus, wild-type E. coli dies or forms a blue colony, whereas the mutant forms a white colony on the X-Gal plate. (B) Schematic showing the crRNA and oligonucleotides used for editing of the E. coli *lacZ* locus. Cleavage sites are indicated by red arrows. The oligonucleotides *lacZ*.lag (59 nt) and *lacZ*.lead (59 nt, reverse and complementary sequence of *lacZ*.lag) were designed to mutate the PAM sequence and introduce a stop codon within the *lacZ* open reading frame. LacZ.del was designed to delete 1,399 bp from the *lacZ* gene. (C) The number and percentage of white colonies of transformants from the electroporation of the indicated pcrRNA plasmids and oligonucleotides (oligo) into E. coli MG1655 expressing recombinase and Cas12a. The transformation efficiency was defined as the total number of CFU generated per transformation. The transformants were plated on X-Gal plates for blue-white screening. The results are the averages of the results from at least two independent experiments, and the error bars depict the standard deviations.

To perform CRISPR-Cas12a-assisted recombineering in E. coli, we first constructed a system composed of two series of plasmids: the pKD46-Cas12a series, which contains a temperature-sensitive pSC101 replicon (31) and expresses the recombination proteins and *Fn*Cpf1 (Cpf1 from Francisella novicida [4]), and the pAC-crRNA series, which contains a p15A replicon (32) and a *sacB* gene for counterselection and expresses crRNA (see Fig. S1 in the supplemental material). To test the efficiency of the system, we selected the *lacZ* gene in MG1655 for gene manipulation, since *lacZ* mutants are easily identified using agar plates supplemented with 5-bromo-4-chloro-3-indolyl-β-d-galactopyranoside (X-Gal) for blue-white colony screening. First, we tested this system by attempting to introduce point mutations into the *lacZ* gene using ssDNA oligonucleotide recombination (Fig. 1A). We designed recombinogenic *lacZ* disruption oligonucleotides targeting the leading and lagging strands of DNA replication, whereby successful recombination introduced a stop codon within the *lacZ* open reading frame (ORF) (Fig. 1B). The oligonucleotides and pAC-crRNA plasmid were electroporated into competent cells harboring pKD46-Cas12a and plated on X-Gal. Only 0.19% of the transformants formed white colonies when the oligonucleotide targeting the *lacZ* lagging strand was cotransformed with pcrRNA-ctrl (without CRISPR-Cas12a targeting). In the presence of CRISPR-Cas12a targeting (using pcrRNA-*lacZ*), approximately 64% of the transformants formed white colonies using an oligonucleotide targeting the leading strand, whereas 81% of the transformants formed white colonies using an oligonucleotide targeting the lagging strand (Fig. 1C). Colony PCR and sequencing confirmed that 15 of 16 white colonies from the lagging-strand transformants and 13 of 16 white colonies from the leading-strand transformants were the correct recombinants. Consistent with a previous study that used CRISPR-Cas9 targeting alone (33, 34), more than 20% of the transformants formed white colonies when pcrRNA-*lacZ* was transformed alone into a strain harboring pKD46-Cas12a (Fig. 1C). Colony PCR showed that the *lacZ* gene had been lost in these mutants, suggesting that homologous recombination might be involved in the formation of these mutants (33).

Next, we tested the system for its ability to generate chromosomal deletions in E. coli. A 79-nucleotide (nt) oligonucleotide with upstream and downstream homology to the area of the deletion was designed to delete 1,399 bp from the *lacZ* gene (Fig. 1B). The oligonucleotide was transformed together with pcrRNA-*lacZ* into E. coli containing pKD46-Cas12a. Approximately one-half of the transformants formed white colonies on X-Gal (Fig. 1C), and colony PCR and sequencing confirmed that 5 of 40 white colonies were the correct recombinants. The other 35 white colonies had the *lacZ* gene deleted by homologous recombination, as previously reported (33, 34). We also performed an experiment to delete the *aroA* gene (1,278 bp) in frame using this system. This experiment yielded approximately 3,000 transformants, of which 4% (3/80) were confirmed to have the desired deletions by colony PCR and sequencing.

Finally, we tested the system for its ability to perform gene replacement using a markerless dsDNA PCR product. *gfp* gene PCR products harboring 45- or 500-bp homology arms for the *aroA* gene were used to replace the *aroA* gene in E. coli. Approximately 0.4% of the transformants were green fluorescent protein (GFP) positive when the 45-bp homology arms were used, whereas approximately 59% of the transformants were GFP positive when the 500-bp homology arms were used. Collectively, the above-described results confirm that our two-plasmid system supports efficient recombineering in E. coli.

### CRISPR-Cas12a-assisted recombineering in Y. pestis.

To determine whether the CRISPR-Cas12a-assisted recombineering system functions in other bacteria, we applied this system to Y. pestis. Point mutations were successfully introduced into two sites (located in *hmsT* and *y4098*) of the chromosome in Y. pestis KIM6+ with recombination efficiencies of 83% and 81%, respectively, using ssDNA oligonucleotides. This result suggests CRISPR-Cas12a-assisted recombineering can be used for genetic manipulation of chromosomal DNA *in* Y. pestis.

**FIG 2:**
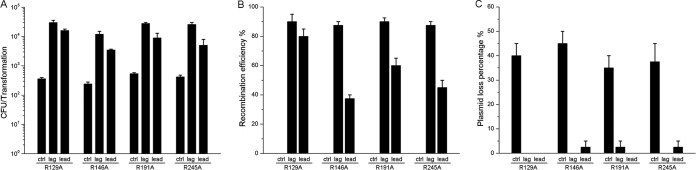
CRISPR-Cas12a-assisted plasmid editing in Y. pestis. Generation of *caf1R* mutations in the pMT plasmid using CRISPR-Cas12a-assisted ssDNA oligonucleotide recombineering. The 59-nt recombinogenic oligonucleotides targeting the lagging strand or the leading strand of DNA replication were utilized for mutation. The transformation efficiency (A), the recombination efficiency (B), and the loss percentage of plasmid (C) are shown as the averages of the results from two independent experiments. ctrl, a control oligonucleotide with no homology to the genome of Y. pestis. Twenty colonies from each transformation were picked to test for plasmid loss and recombination by colony PCR and sequencing.

Native plasmids are usually important for antibiotic resistance, symbiosis, metabolism, and virulence in bacteria. To the best of our knowledge, CRISPR-assisted recombineering has not been applied for genetic manipulation of native plasmids in bacteria. Wild-type Y. pestis harbors three plasmids (pPCP1, pMT1, and pCD1) that are important for the virulence and environmental adaptation of the pathogen (35). To test whether genetic manipulation could be conducted on native plasmids, we performed ssDNA oligonucleotide recombination experiments to mutate the *caf1R* gene located in the pMT1 plasmid using the CRISPR-Cas12a-assisted recombineering system. Four arginine codons were mutated to alanine codons individually in the *caf1R* gene with recombination rates of approximately 90% when oligonucleotides targeting the lagging strand were used (Fig. 2). Furthermore, the pMT1 plasmid was cured from Y. pestis in approximately 40% of transformants when the control oligonucleotide was used (Fig. 2), indicating that CRISPR-Cas12a could be used to cure native plasmids (36). We conclude that the CRISPR-Cas12a-assisted recombineering is a useful method for genetic manipulation of chromosomal and plasmid DNA in Y. pestis.

### Evaluation of Cas12a activity in M. smegmatis.

The next goal of this study was to establish CRISPR-Cas12a selection in mycobacteria to enable high-efficiency recombineering. To evaluate the feasibility of this goal, we cloned and expressed *Fn*Cpf1 in mycobacteria. The gene encoding Cas12a was modified to optimize expression in mycobacteria (Fig. S2). Although Cas12a is an exogenous protein, induced expression of Cas12a (up to 100 ng · ml^−1^ anhydrotetracycline [ATc]) did not strongly affect the growth of M. smegmatis (Fig. S3A). Next, we performed plasmid interference assays to test *Fn*Cpf1 activity in mycobacteria (37). In this approach, expression of a Cas endonuclease and a crRNA on a transformed plasmid results in cleavage of the target plasmid, which is reflected as a decrease in the number of transformants. For this purpose, we constructed a plasmid for coexpression of Cas12a and a crRNA targeting the *gfp* gene (pCpf1-gfp) or a crRNA not targeting the *gfp* gene (pCpf1-ctrl). M. smegmatis harboring pCpf1-gfp or pCpf1-ctrl was transformed with pJV53 (without *gfp*) or pJV53-GFP (with *gfp*). Expression of Cas12a and the *gfp*-specific crRNA significantly decreased the number of transformants with pJV53-GFP but not the number of transformants with pJV53, whereas expression of Cas12a and the nonspecific crRNA did not affect the transformation efficiency of either pJV53-GFP or pJV53 (Fig. S3B). Taken together, these results suggest that *Fn*Cpf1 is biologically active and can mediate targeted DNA interference in M. smegmatis.

### Genetic manipulation in M. smegmatis using CRISPR-Cas12a-assisted ssDNA oligonucleotide recombineering.

**FIG 3:**
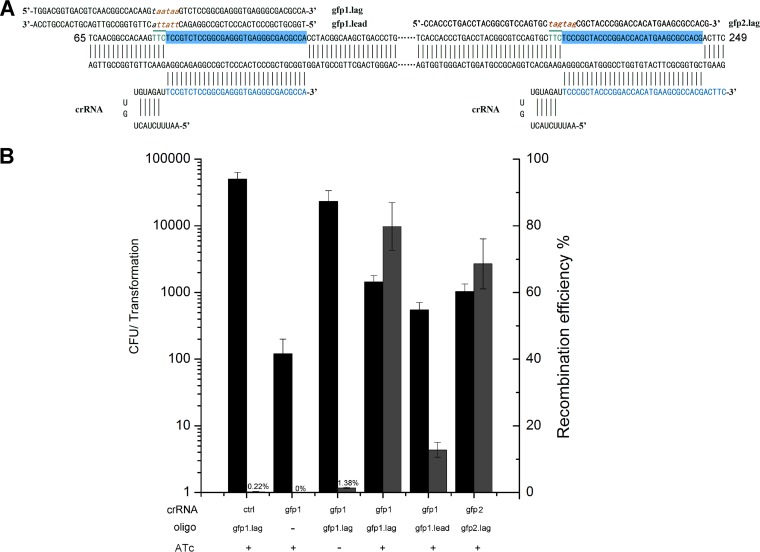
CRISPR-Cas12a-assisted single-stranded oligonucleotide recombineering in M. smegmatis. (A) Schematic showing the sequence of the *gfp*-targeting crRNA and oligonucleotides used for recombineering. This region corresponds to nucleotides 65 to 249 of the *gfp* ORF. The 60-nt recombinogenic oligonucleotides targeting the lagging strand (gfp1.lag and gfp2.lag) or targeting the leading strand (gfp1.lead) of DNA replication were utilized to disrupt the *gfp* gene by introducing changes in five consecutive base pairs (shown in brown) to generate two consecutive in-frame stop codons (lowercase and italic). The green and top-lined sequences represent the PAM. (B) Transformation and *gfp* recombination efficiency resulting from electroporation of the indicated pcrRNA plasmids and oligonucleotides into M. smegmatis expressing recombinase and Cas12a (*Fn*Cpf1). The transformation efficiency was defined as the total number of CFU generated per transformation, and the recombination efficiency was measured by determining the proportion of GFP-negative colonies. ATc (50 ng/ml) was added to the agar to induce Cas12a expression. Results are the averages of the results from at least two independent experiments, and the error bars depict the standard deviations.

To determine whether the CRISPR-Cas12a system could be coupled to oligonucleotide recombineering in mycobacteria, we constructed a modified M. smegmatis strain in which the *gfp* gene was inserted into the chromosome at the *Ms5635–Ms5634* locus. We also constructed a new two-plasmid system (Fig. S4) as follows: pJV53-Cpf1, which expresses *Fn*Cpf1 and the recombination proteins gp60 and gp61 (18), and the pCR series (pCR-Hyg and pCR-Zeo), which contain a temperature-sensitive replicon (38, 39) and expresses crRNAs. This two-plasmid system was tested for its ability to generate targeted recombinants. For this purpose, we designed recombinogenic *gfp* disruption oligonucleotides (Fig. 3A) targeting the lagging strands of the DNA replication fork; successful recombination with these oligonucleotides would lead to mutation of the PAM sequence and introduce a stop codon within the *gfp* open reading frame (ORF; Fig. 3A), which could easily be confirmed by detecting loss of the GFP signal. As shown in Fig. 3B, cotransformation of pcrRNA-gfp1 and the lagging-strand oligonucleotide generated approximately 10^3^ colonies, of which 80% were GFP negative. Consistent with the previous observation that an oligonucleotide targeting the lagging strand produces more recombinants than an oligonucleotide targeting the leading strand (40), cotransformation of pcrRNA-gfp1 and the leading-strand oligonucleotide generated fewer GFP-negative recombinants (Fig. 3B). In addition, another mutation in the *gfp* gene was generated with similar efficiency using this system (Fig. 3). Finally, we generated two mutations (T49V and C86S) in *Ms1521*, an essential gene in M. smegmatis, with 87.5% and 60% recombination rates using CRISPR-Cas12a-assisted ssDNA recombineering.

**FIG 4:**
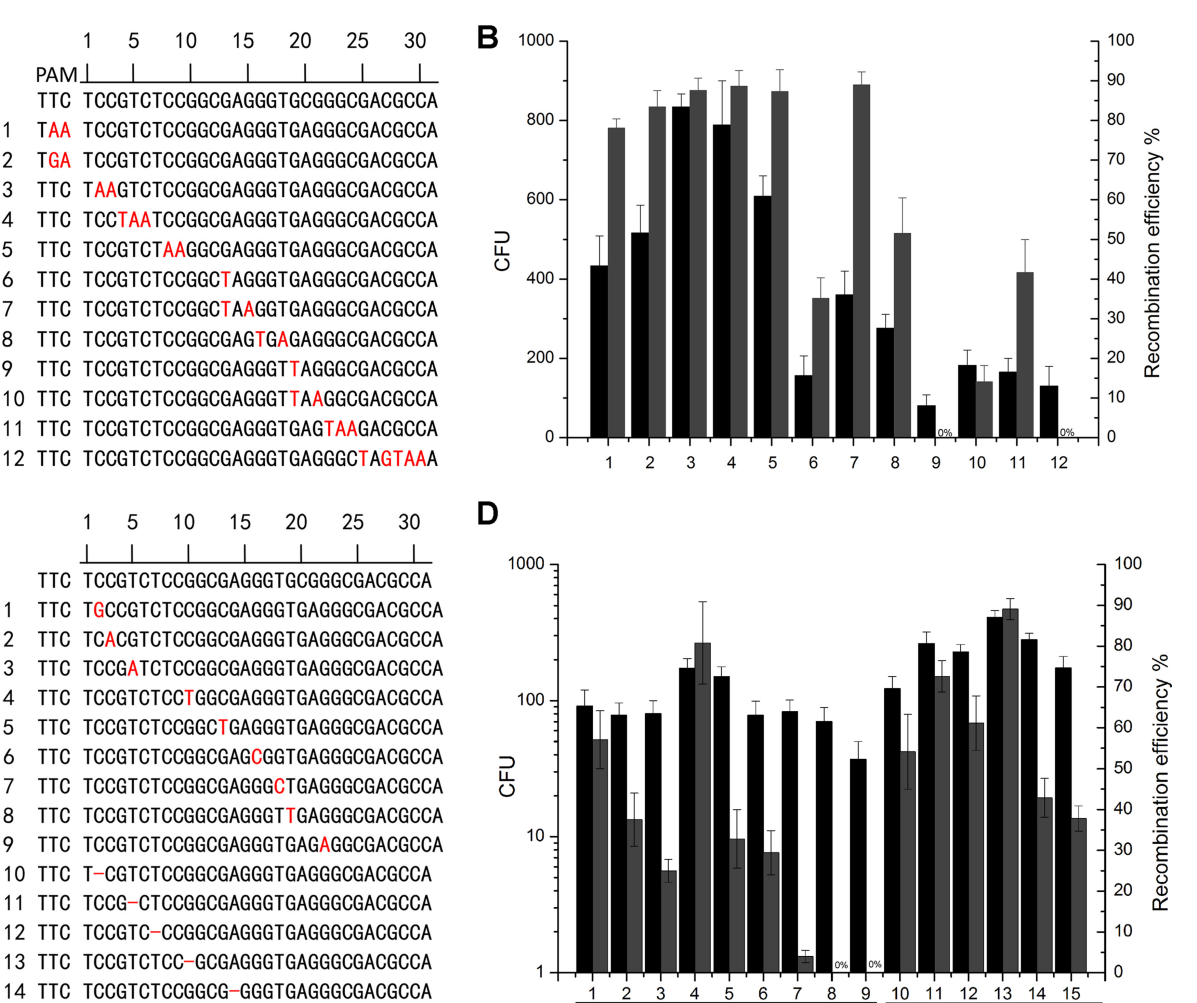
Generation of subtle gene mutations using CRISPR-Cas12a-assisted ssDNA oligonucleotide recombineering in M. smegmatis. (A) Schematic showing the sequences of the point mutations generated in the *gfp* gene. Mutated sequences are shown in red. (B) Generation of point mutations described in panel A using CRISPR-Cas12a-assisted ssDNA oligonucleotide recombineering. (C) Schematic showing the sequences of 1-bp frameshifts generated in the *gfp* gene. Inserted sequences are shown in red, and the dashed line indicates the deleted sequence. (D) Generation of 1-bp insertions or deletions described in panel C using CRISPR-Cas12a-assisted ssDNA oligonucleotide recombineering. Transformation efficiency was defined as the total number of CFU generated per transformation, and recombination efficiency was measured by determining the proportion of GFP-negative colonies. Results are the averages of the results from at least two independent experiments, and the error bars depict the standard deviations.

To bind a target gene, Cas12a recognizes thymidine-rich PAM sequences and the targeting crRNA. Consequently, point mutations can be introduced into the PAM- and crRNA-targeting regions using CRISPR-Cas12a-assisted ssDNA oligonucleotide recombineering (26, 41, 42). We sought to determine the sites that were susceptible to mutation by testing the recombination efficiency of a series of lagging-strand oligonucleotides designed to introduce a stop codon in the *gfp* gene by mutation of one to three nucleotides (Fig. 4A). A 2- or 3-bp mutation was successfully introduced into the PAM- or crRNA-targeting region (Fig. 4B). Consistent with a previous report showing that a 16-nt guide sequence is required to achieve detectable DNA cleavage and a 18-nt guide sequence is required to achieve efficient DNA cleavage (4), a 1-bp mutation was introduced at position 13 of the spacer region but not at position 19 (Fig. 4B). Additionally, consistent with a previous report that the crRNA of Cas12a is 24 nt (4, 8), a mutation of several base pairs was achieved at positions 19 to 21 or 22 to 24 but not at positions 25 to 30 (Fig. 4B). To ascertain site-specific susceptibility to mutation, we designed a series of lagging-strand oligonucleotides to introduce a frameshift mutation into the *gfp* gene by insertion or deletion of one nucleotide (Fig. 4C). We found that a 1-bp insertion or deletion was efficiently generated at positions 1 to 18 of the protospacer sequence but not at positions 19 and 22 (Fig. 4D). Taken together, these experiments suggest that our system can efficiently introduce point mutations into PAM- and crRNA-targeting regions in M. smegmatis.

**FIG 5:**
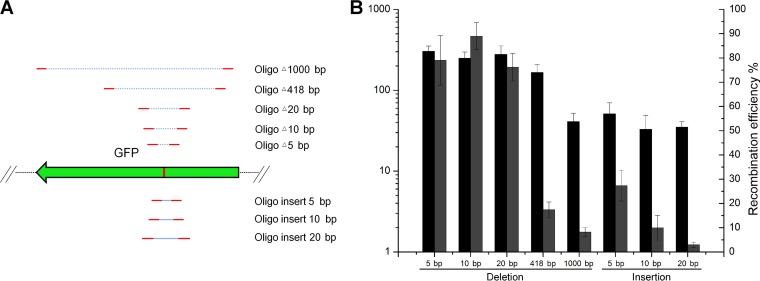
Gene deletion and insertion using CRISPR-Cas12a-assisted single-stranded oligonucleotide recombineering in M. smegmatis. (A) Schematic of gene deletions and insertions generated in the *gfp* gene using CRISPR-Cas12a-assisted single-stranded oligonucleotide recombineering in M. smegmatis. (B) Generation of deletions and insertions shown in panel A using CRISPR-Cas12a-assisted single-stranded oligonucleotide recombineering in M. smegmatis. Introduction of a 5-, 10-, 20-, 418-, or 1,000-bp deletion or 5-, 10-, or 20-bp insertion in the *gfp* gene used CRISPR-Cas12a-assisted single-stranded oligonucleotide recombineering. The transformation efficiency was defined as the total number of CFU generated per transformation, and recombination efficiency was measured by determining the proportion of GFP-negative colonies. Results are the averages of the results from at least two independent experiments, and error bars depict standard deviations.

Next, we tested whether CRISPR-Cas12a-assisted recombineering could generate gene deletions in mycobacteria. For this purpose, we designed 59- or 79-mer oligonucleotides, which yielded a 5-, 10-, 20-, 418-, or 1,000-bp deletion when incorporated into the chromosome, thereby disrupting the protospacer sequence in the *gfp* gene (Fig. 5A). Deletions of 5, 10, or 20 bp were easily generated using 59-mer oligonucleotides, and 70 to 80% of the transformants were recombinants (Fig. 5B). However, the recombineering efficiency decreased as the size of the deleted fragment increased. Deletion of a 1,000-bp fragment could not be achieved using the 59-mer oligonucleotide, although the efficiencies of deletion of 418- and 1,000-bp fragments from the *gfp* gene were 17.4% and 8.2%, respectively, when 79-mer oligonucleotides were used. These results demonstrate that CRISPR-Cas12a-assisted ssDNA recombineering is a feasible method for the construction of gene deletions in M. smegmatis.

Next, we investigated whether CRISPR-Cas12a-assisted recombineering allowed the isolation of oligonucleotide-mediated chromosome insertions. A series of oligonucleotides were designed to yield 5-, 10-, or 20-bp insertions and to disrupt the protospacer sequence in the *gfp* gene (Fig. 5A). The efficiency of the oligonucleotide-mediated chromosome insertion was strongly affected by the size of the insertion fragment. Approximately 27% of the transformants contained 5-bp insertions, and 10% had 10-bp insertions, whereas only 3.1% had a 20-bp insertion (Fig. 5B). Together, the results presented in this section show that this system can generate short insertions using ssDNA oligonucleotide recombineering in M. smegmatis.

### Markerless gene manipulation in M. smegmatis using CRISPR-Cas12a-assisted dsDNA recombineering.

**FIG 6:**
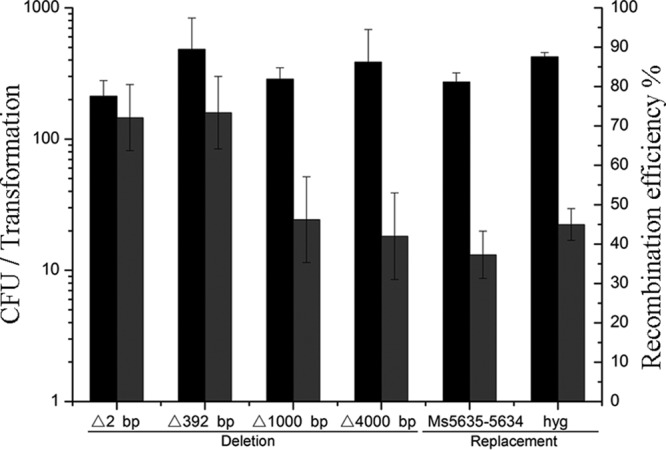
Double-stranded DNA recombineering assisted by CRISPR-Cas12a. Deletions of 2, 392, 1,000, or 4,000 bp were introduced into M. smegmatis chromosomal DNA using approximately 1-kb double-stranded DNA fragments with induced Cas12a. The *gfp* gene was replaced with a dsDNA PCR fragment containing the Hyg resistance gene or *Ms5635–Ms5634* and its flanking region with induced Cas12a. Diagrams of the above gene deletions and replacements are shown in Fig. S5. Transformation efficiency was defined as the total number of CFU generated per transformation, and recombination efficiency was measured by determining the proportion of GFP-negative colonies. Results are the averages of the results from at least two independent experiments, and error bars depict standard deviations.

We next attempted to use the Cas12a-assisted recombineering system to perform chromosomal gene deletion in M. smegmatis with a markerless dsDNA template. We targeted the same *gfp* gene region used in the oligonucleotide recombineering experiments described above. Four dsDNA fragments containing 2-, 392-, 1,000-, or 4,000-bp deletions in the *gfp* region were constructed and cotransformed with pcrRNA-gfp1. As shown in Fig. 6, markerless gene deletions up to 4 kb were obtained with high efficiency. We also investigated whether dsDNA PCR products could be used in this system for gene manipulation in M. smegmatis. The *gfp* gene was successfully replaced by *Ms5635–Ms5634* and the *hyg* antibiotic resistance gene using PCR products as homology recombination fragments (Fig. 6). Taken together, these experiments suggest that CRISPR-Cas12a-assisted dsDNA recombineering is an efficient method for markerless gene manipulation.

The introduction of multiple mutations is often necessary to study the functions of redundant genes. For example, four pairs of toxin-antitoxin (TA) genes (*Ms1277–Ms1278*, *Ms1283–Ms1284*, *Ms4447–Ms4448*, and *Ms5635–Ms5634*) are present in M. smegmatis. To sequentially delete these genes, crRNA sequences targeting *Ms1278* (pYC1009) and *Ms4447* (pYC983) were cloned into pCR-Hyg, while crRNA sequences targeting *Ms1283* (pYC1010) and *Ms5635* (pYC1011) were cloned into pCR-Zeo. Recombineering DNA fragments were also constructed for the in-frame deletion of the TA genes and transformed in combination with the corresponding crRNA-expressing plasmid to sequentially delete the four TA genes. The resulting transformants were selected and verified by PCR at each step (Fig. S6). The recombineering efficiencies for deletion of the four TA genes were 62%, 53%, 45%, and 47%, respectively. The four in-frame scarless deletions were obtained in approximately 5 weeks, compared to the >10 weeks normally required using suicide vector-based allelic replacement (43). Taken together, these results demonstrate that our novel system is a time-efficient approach for the construction of multiple markerless and scarless mutations in M. smegmatis.

## DISCUSSION

Recombineering is a powerful method that was developed in the last decade to introduce precise genetic changes into bacterial genomes. Recently, CRISPR-Cas9 was used to facilitate recombineering that simplifies genetic manipulation methods and abolishes reliance on antibiotic markers (22–27, 44). Genetic manipulation in mycobacteria is time-consuming, especially if multiple changes are needed. Therefore, more efficient molecular tools are required to meet the increasing need for more diverse types of genetic manipulation. In this study, we described the development of a system that couples CRISPR-Cas12a genome editing with recombineering to allow efficient genetic manipulation in E. coli, Y. pestis, and M. smegmatis.

ssDNA recombineering has been used to generate point mutations, deletions, and small insertions in E. coli (45), as well as to introduce mutations into mycobacteria (40). However, the recombination efficiency is low, and isolating the recombinants is difficult, which hinders the practical application of this method. CRISPR-Cas9 has been successfully used in ssDNA recombineering to isolate recombinants in E. coli and L. reuteri (25–27). Very recently, Jiang and colleagues showed that CRISPR-Cas12a-assisted genome editing can be used in Corynebacterium glutamicum (15). We showed here that ssDNA recombineering assisted by the CRISPR-Cas12a system can efficiently generate point mutations and deletions in E. coli, Y. pestis, and M. smegmatis (Fig. 1 to 4). More interestingly, this method can be used to insert DNA fragments of up to 20 bp into the chromosome (Fig. 5). Thus, Cas12a-assisted ssDNA recombineering could be used to insert a tag-encoding sequence, such as DAS+4 (12 bp [46]) or His_6_ (18 bp), into a specific gene on the chromosome. Because this method does not require the construction of a dsDNA fragment for recombination, Cas12a-assisted ssDNA recombineering is time-efficient and cost-effective for bacterial genome editing.

Consistent with a previous report showing that Cas12a provides highly specific gene targeting in human and murine cells (47–49), Cas12a was also highly specific in mycobacteria. Cas12a is sensitive to mismatched crRNA nucleotides at positions 1 to 24 of the targeting region but not to mismatches at positions 25 to 31. This finding is supported by the observation that a 2- or 3-bp mutation can be introduced into the PAM- or crRNA-targeting region (positions 1 to 24), whereas mutations cannot be introduced into positions 25 to 31 (Fig. 4). Additionally, we did not detect any mutations in the absence of ssDNA when both Cas12a and crRNA were expressed (Fig. 3B). Taken together, these results suggest that Cas12a-assisted recombineering is highly specific and confirm that nonhomologous end-joining (NHEJ) mutants are unlikely to be generated in M. smegmatis using this method.

Cas12a-assisted genome editing has broadened the application of the CRISPR-Cas system in bacteria. First, Cas12a is an alternative to Cas9 when Cas9 is toxic in an organism (15). Second, Cas9 recognizes an NGG PAM sequence, whereas Cas12a recognizes a YTN (CTN or TTN) PAM sequence (10). Therefore, Cas12a and Cas9 recognize different target sites, which allows for more precise selection of the cleavage target; this is especially important for the generation of point mutations in the genome. Finally, the Cas12a cleavage site is 18 and 23 positions distal to the PAM sequence; thus, an indel resulting from NHEJ would not disrupt the PAM, and the resulting sequence could be cleaved, which could increase the likelihood of recovering homologous recombinants and decrease the likelihood of recovering NHEJ-mediated mutants. This is potentially important given that NHEJ is present and functional in M. smegmatis (23, 50, 51). However, we did not detect any NHEJ in M. smegmatis using Cas12a-assisted recombineering.

Our method is especially useful for the introduction of multiple genetic mutations into bacteria. In this regard, it has several advantages: (i) following recombination, the helper plasmids can be easily cured because they contain either a temperature replicon or the *sacB* counterselection gene; (ii) crRNA plasmids containing different resistance cassettes can be used in alternation when multiple genes must be deleted sequentially, thereby decreasing the time required to cure the crRNA plasmid in multiple-gene deletions; (iii) the use of an antibiotic gene as a selection marker, whose removal usually requires an additional step, is unnecessary; and (iv) the creation of chromosome scar sites (e.g., LoxP recognition sites) is avoided. The presence of multiple scars in the chromosome can lead to chromosomal rearrangements or deletions resulting from recombination between scars.

Overall, the approach described in this report demonstrates that coupling the recombination system with the CRISPR-Cas12a system simplifies the construction of scarless genome mutations, especially multiple mutations, in bacteria. Given that the Che9c recombination system has been successfully used in M. tuberculosis (18), we postulate that this methodology could also be applied to other mycobacteria, including M. tuberculosis, although this still needs to be tested.

## MATERIALS AND METHODS

### Strains, media, and growth conditions.

E. coli MG1655, Y. pestis KIM6+, and M. smegmatis mc^2^155 were used in this study. E. coli and Y. pestis were grown in LB medium supplemented with appropriate antibiotics (25 μg/ml kanamycin, 100 μg/ml ampicillin, or 30 μg/ml chloramphenicol). M. smegmatis was grown in Middlebrook 7H9 broth (Difco) supplemented with 0.05% Tween 80 and 0.2% glycerol or on Middlebrook 7H10 agar supplemented with the appropriate antibiotic (25 μg/ml kanamycin, 50 μg/ml hygromycin, or 50 μg/ml zeocin). Appropriate concentrations of anhydrotetracycline (ATc) were added to the M. smegmatis cultures when necessary. To facilitate the screening of recombinants in M. smegmatis, a GFP reporter gene was inserted into the *Ms5635–Ms5634* locus of the chromosome using recombineering (52).

### Plasmids.

The *Fn*Cpf1 open reading frame (ORF) sequence was cloned into pKD46 (13) using Gibson cloning to yield pKD46-Cas12a series plasmids (Fig. S1A). The pKD46-Cas12a series plasmids, which contain a temperature-sensitive replicon, can be cured at 42°C. The pre-crRNA cassette containing the *gfp* gene was commercially synthesized and cloned together with the *sacB* gene into the modified pACYC184 vector to yield pAC-crRNA series plasmids (Fig. S1). The *gfp* gene, which is flanked by BpmI and BsaI restriction enzyme sites, was used as a selection marker for protospacer cloning (Fig. S1). Two complementary oligonucleotides containing the target sequence adjacent to 5′-YTN-3′ were synthesized, annealed to yield a protospacer cassette with BpmI or BsaI overhangs at the 5′ and 3′ ends, respectively, and then cloned into the pAC-crRNA plasmid (Fig. S1). The research presented in this study was conducted using the pKD46-Cas12a-Amp and pAC-crRNA-Cm plasmids. pKD46-Cas12a-Cm and the other pAC-crRNA series plasmids were also tested and yielded results similar to those obtained using npKD46-Cas12a-Amp and pAC-crRNA-Cm plasmids (data not shown).

A codon-optimized *Fn*Cpf1 ORF sequence (optimized with JCat) (53) (Fig. S2) under the control of the *P_myc1_tetO* promoter was commercially synthesized (GENEWIZ) and cloned into pMV261 and pJV53 to yield pMV261-Cpf1 and pJV53-Cpf1, respectively (Fig. S4A). The pre-crRNA cassette was commercially synthesized and cloned into pSL003 (54) to yield pCR-Zeo (Fig. S4B). The hygromycin resistance gene was amplified by PCR from pSL002 and cloned into pCR-Zeo to replace the zeocin resistance gene, yielding pCR-Hyg (Fig. S4B). The Cas12a gene was amplified by PCR from pMV261-Cpf1 and then ligated into pcrRNA-ctrl and pcrRNA-gfp1 digested with KpnI and NotI, to yield pCpf1-ctrl and pCpf1-gfp, respectively. To mutate a particular gene in mycobacteria, two complementary oligonucleotides containing the target sequence adjacent to ′-YTN-3′ were synthesized, annealed to yield a protospacer cassette with BpmI and HindIII overhangs at the 5′ and 3′ ends, respectively, and then cloned into pCR-Zeo or pCR-Hyg. All plasmids constructed in this study are listed in Table S1. The oligonucleotides used in this study are listed in Table S2.

### M. smegmatis growth assay.

M. smegmatis cells harboring pMV261 or pMV261-Cpf1 were inoculated into 3 ml of 7H9 broth supplemented with kanamycin and grown overnight with shaking at 37°C. The overnight cultures were diluted to an optical density at 600 nm (OD_600_) of 0.02 in 50 ml of 7H9 broth supplemented with kanamycin and appropriate concentrations of ATc. These cultures were grown overnight with shaking at 37°C, and samples were taken at appropriate time points to determine the OD_600_.

### Plasmid interference assay.

The plasmid interference assay was carried out as previously reported, with minor modifications (37). Briefly, M. smegmatis mc^2^155 harboring pCpf1-ctrl or pCpf1-gfp was transformed with pJV53 or pJV53-GFP, respectively. The transformants were plated onto 7H10 medium supplemented with or without 50 ng/ml ATc and then grown for 3 days at 37°C. The colonies were counted to calculate the CFU.

### Preparation of recombinogenic DNA.

For the ssDNA oligonucleotide recombineering experiments, the recombinogenic oligonucleotides were synthesized, and mutations were introduced into the middle of the oligonucleotide sequences with at least 25 nt of sequence identity on both sides of the mutation site. The leading and lagging strands of the bacterial chromosomes were determined using cumulative skew diagrams (55).

For the dsDNA recombineering experiments, the *gfp* gene was amplified from pAcGFP1 vector using primers with 45-nt homology regions of *aroA* to generate recombinogenic dsDNA products for *aroA* replacement in E. coli. To generate dsDNA homologous arms for the deletion of 2, 392, or 1,000 bp from the *gfp* gene in M. smegmatis, the *Ms5635–Ms5634*::*gfp* cassette with flanking regions was amplified by PCR and inserted into pUC19 to yield the plasmid pYC847. Then, pYC847 was used as the template for inverse PCR with appropriate primer sets to generate plasmids containing dsDNA homologous arms. To generate dsDNA homologous arms for the 4,000-bp deletion, a 539-bp DNA fragment downstream of *Ms5634* and a 596-bp DNA fragment upstream of *Ms5635* were amplified by PCR and cloned into pUC19 to yield pYC848. These plasmids were digested with KpnI and SphI, and the digested fragments were gel purified. pYC710, pYC711, pYC799, and pYC738 were used as templates to generate pYC984, pYC985, pYC986, and pYC987, which contain the dsDNA homologous arms for the toxin-antitoxin systems *Ms1277–Ms1278*, *Ms1283–Ms1284*, *Ms4447–Ms4448*, and *Ms5635–Ms5634*, respectively. The dsDNA homologous arms generated from these new plasmids were digested with HindIII and KpnI. *Ms5635–Ms5634*, the *hyg* resistance gene, and their flanking regions were amplified by PCR from the chromosomes of wild-type M. smegmatis and the M. smegmatis *Ms5635–Ms5634*::*hyg* mutant, respectively, and used as recombinogenic fragments for the replacement of the *gfp* gene. The primers used in this study are listed in Table S2.

### Cas12a-assisted genome editing.

Competent cells of E. coli, Y. pestis, and M. smegmatis were prepared as previously described (17, 52, 56). For ssDNA oligonucleotide recombineering, approximately 500 ng of recombinogenic or nonrecombinogenic oligonucleotides and 100 ng of the crRNA expression plasmid were mixed and electroporated into competent cells. For dsDNA gene deletion, 700 ng of gel-purified restriction-digested product or PCR product and 100 ng of the crRNA-expressing plasmid were mixed and electroporated into competent cells. For E. coli and Y. pestis, the electroporated cells were plated and grown on LB agar supplemented with appropriate antibiotics overnight at 30°C. Recombination of *lacZ* was assessed by examination for the formation of white colonies and then confirmed by PCR and sequencing analysis. Recombination in Y. pestis was analyzed and verified by PCR amplification and sequencing. Plasmid-free colonies were obtained by incubating the cells in LB culture medium supplemented with sucrose at 42°C. For M. smegmatis, cells were recovered after incubation in 1 ml of 7H9 broth with 10 ng/ml ATc for 4 h at 30°C at 200 rpm and then plated on 7H10 agar supplemented with the appropriate antibiotics and 50 ng/ml ATc. After growth for 4 days at 30°C, the plates contained normal-size and tiny transformant colonies. The normal colonies were counted and used to calculate the transformation and recombination efficiencies. Recombination of *gfp* was assessed by examining for the loss of the GFP signal, and then each time, at least 5 GFP-negative recombinants were picked for PCR and sequencing analysis, and all of the tested colonies were confirmed to be desired recombinants. Recombination involving other genes was assessed by PCR and sequencing. To cure the helper plasmids from the M. smegmatis recombinant, the right recombinant colony was picked and grown overnight at 37°C in 7H9 medium without antibiotic. The overnight culture was diluted 1:500 into 7H9 medium and grown for 24 h at 37°C. The resultant cultures were diluted, plated, and grown for 3 days on 7H10 plates and then tested for the loss of plasmids using replica streaking on the plate with or without appropriate antibiotics.

## Supplementary Material

Supplemental material
