# Supplementary material for: CRISPR-Cas12a-Assisted Recombineering in Bacteria
Source: Appl Environ Microbiol. 2017 Aug 17;83(17):e00947-17. doi: 10.1128/AEM.00947-17 (PMC5561284; doi:10.1128/AEM.00947-17)
Supplement: Supplemental material [file supp_83_17_e00947-17__index.html]

Supplemental material 

# CRISPR-Cas12a-Assisted Recombineering in Bacteria

## Supplemental material

- Supplemental file 1 -

  CRISPR-Cpf1-assisted pKD46-based recombineering system (Fig. S1), analysis of FnCpf1 sequence codon-optimized for expression in mycobacteria (Fig. S2), functional analysis of FnCpf1 in *M. smegmatis* (Fig. S3), CRISPR-Cpf1-assisted pJV53-based recombineering system (Fig. S4), diagrams of gene deletions and replacements in Fig. 6 (Fig. S5), sequential deletion of four TA genes using CRISPR-Cpf1–assisted recombineering (Fig. S6), and plasmids (Table S1) and oligonucleotides (Table S2) used in the study.

  PDF, 1.5M
