# Supplementary material for: CRISPR-Cas12a-Assisted Recombineering in Bacteria
Source: Appl Environ Microbiol. 2017 Aug 17;83(17):e00947-17. doi: 10.1128/AEM.00947-17 (PMC5561284; doi:10.1128/AEM.00947-17)
Supplement: Supplemental material [file AEM.00947-17_zam999118011s1.pdf]

Supplemental Material

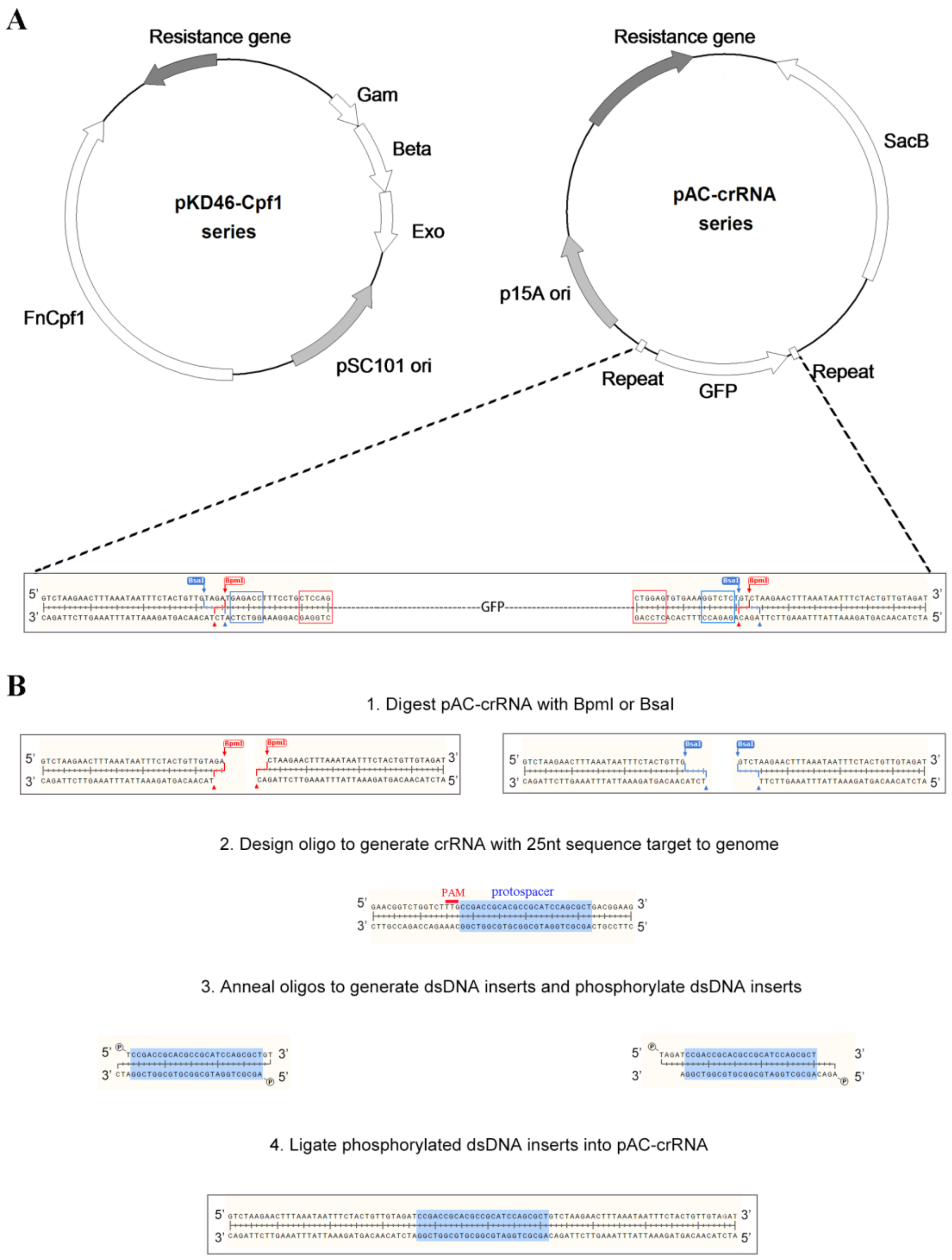

Figure S1. CRISPR-Cpf1-assisted pKD46 based recombineering system. (A) Schematic of the pKD46-Cpf1 series (GenBank MF287367) and pAC-crRNA series (GenBank MF287368 for pAC-crRNA-Cm, GenBank MF287369 for pAC-crRNA-Str, GenBank MF287370 for pAC-crRNA-Km) plasmids. The pKD46-Cpf1 series plasmids contain either an ampicillin or kanamycin resistance genes. The pAC-crRNA series plasmids contain chloramphenicol, kanamycin or streptomycin resistance genes. The crRNA cassette contains a *gfp* reporter gene flanking with BpmI and BsaI restriction enzyme sites to facilitate cloning of the pre-crRNA. (B) Cloning strategy for the construction of plasmids to express crRNAs targeting genes of interest. The cloning strategy for the crRNA sequences targeting *lacZ* gene is shown blow as an example. The same strategy was used to construct other crRNA plasmids used in *E. coli* and *Y. pestis*.

Optimized	1	ATGTCGATCTACCAAGAGTTCGTGAATAAGTATAGCCTGAGCAAGACCTTGCGGTTCGAGTTGATCCCGC
Original	1	ATGTCAATTTATCAAGAATTTGTTAATAAATATAGTTTAAGTAAAACTCTAAGATTTGAGTTAATCCCAC
Optimized	71	AGGGCAAAACCTTGGAAAACATTAAGGCGCGCGGGCTGATCCTGGATGATGAGAAGCGGGCCAAGGACTA
Original	71	AGGGTAAAACACTTGAAAACATAAAAGCAAGAGGTTTGATTTTAGATGATGAGAAAAGAGCTAAAGACTA
Optimized	141	TAAAAAGGCCAAGCAGATTATCGATAAATATCATCAGTTCTTCATCGAGGAAATCCTGTCGAGCGTGTGC
Original	141	CAAAAAGGCTAAACAAATAATTGATAAATATCATCAGTTTTTTATAGAGGAGATATTAAGTTCGGTTTGT
Optimized	211	ATCAGCGAAGACTTGTTGCAGAACTATAGCGACGTGTACTTCAAATTGAAAAAGTCGGACGACGACAATC
Original	211	ATTAGCGAAGATTTATTACAAAACTATTCTGATGTTTATTTTAAACTTAAAAAGAGTGATGATGATAATC
Optimized	281	TGCAAAAAGACTTCAAGTCGGCCAAAGACACCATCAAGAAACAGATTTCGGAGTACATCAAGGACTCGGA
Original	281	TACAAAAAGATTTTAAAAGTGCAAAAGATACGATAAAGAAACAAATATCTGAATATATAAAGGACTCAGA
Optimized	351	AAAGTTCAAAAACCTGTTCAACCAGAACTTGATCGACGCGAAAAAGGGCCAGGAAAGCGATTTGATCTTG
Original	351	GAAATTTAAGAATTTGTTTAATCAAAACCTTATCGATGCTAAAAAAGGGCAAGAGTCAGATTTAATTCTA
Optimized	421	TGGCTGAAGCAGTCGAAGGATAATGGCATTGAGTTGTTCAAGGCCAACTCGGACATTACGGACATTGACG
Original	421	TGGCTAAAGCAATCTAAGGATAATGGTATAGAACTATTTAAAGCCAATAGTGATATCACAGATATAGATG
Optimized	491	AGGCCCTGGAAATCATTAAATCGTTTAAGGGCTGGACCACGTACTTCAAGGGCTTCCATGAGAACCGGAA
Original	491	AGGCGTTAGAAATAATCAAATCTTTTAAAGGTTGGACAACTTATTTTAAGGGTTTTCATGAAAATAGAAA
Optimized	561	AAACGTGTATTCGTCGAACGACATTCCCACGTCGATCATCTACCGGATCGTCGATGACAACTTGCCCAAG
Original	561	AAATGTTTATAGTAGCAATGATATTCCTACATCTATTATTTATAGGATAGTAGATGATAATTTGCCTAAA
Optimized	631	TTCTTGGAAAATAAAGCGAAATACGAGAGCTTGAAGGACAAAGCGCCGGAGGCCATTAACTATGAGCAGA
Original	631	TTTCTAGAAAATAAAGCTAAGTATGAGAGTTTAAAAGACAAAGCTCCAGAAGCTATAAACTATGAACAAA
Optimized	701	TCAAGAAGGACCTGGCCGAAGAGCTGACCTTTGACATTGACTACAAGACCAGCGAGGTCAATCAGCGGGT
Original	701	TTAAAAAGATTTGGCAGAAGAGCTAACCTTTGATATTGACTACAAAACATCTGAAGTTAATCAAAGAGT
Optimized	771	CTTCTCGTTGGATGAGGTCTTTGAAATCGCCAATTTCAACAACTATTTGAACCAGAGCGGCATCACCAAA
Original	771	TTTTTCACTTGATGAAGTTTTTGAGATAGCAAACTTTAATAATTATCTAAATCAAAGTGGTATTACTAAA
Optimized	841	TTTAATACCATTATCGGCGGGAAGTTCGTCAACGGCGAGAACACGAAGCGGAAGGGGATCAACGAATACA
Original	841	TTTAATACTATTATTGGTGGTAAATTTGTAAATGGTGAAAATACAAAGAGAAAAGGTATAAATGAATATA
Optimized	911	TCAATCTGTACTCGCAGCAGATCAACGACAAGACCCTGAAAAAGTACAAGATGAGCGTGCTGTTCAAGCA
Original	911	TAAATCTATACTCACAGCAAATAAATGATAAAACACTCAAAAAATATAAAATGAGTGTTTTATTTAAGCA
Optimized	981	AATTCTGAGCGACACCGAGAGCAAGTCGTTCGTCATTGACAAGCTGGAGGATGACAGCGATGTGGTCACC
Original	981	AATTTTAAGTGATACAGAATCTAAATCTTTTGTAATTGATAAGTTAGAAGATGATAGTGATGTAGTTACA
Optimized	1051	ACGATGCAGTCGTTTTACGAGCAAATCGCGGCCTTCAAGACCGTGGAGGAAAAGAGCATCAAGGAAACCC
Original	1051	ACGATGCAAAGTTTTTATGAGCAAATAGCAGCTTTTAAAACAGTAGAAGAAAAATCTATTAAAGAAACAC
Optimized	1121	TGAGCCTGCTGTTCGATGATCTGAAGGCGCAGAAGTTGGATCTGTCGAAAATCTACTTCAAGAACGATAA
Original	1121	TATCTTTATTATTTGATGATTTAAAAGCTCAAAAACTTGATTTGAGTAAAATTTATTTTAAAAATGATAA
Optimized	1191	GTCGCTGACCGACCTGAGCCAACAGGTCTTCGATGATTACAGCGTCATCGGGACCGCCGTCCTGGAGTAC
Original	1191	ATCTCTTACTGATCTATCACAACAAGTTTTTGATGATTATAGTGTTATTGGTACAGCGGTACTAGAATAT
Optimized	1261	ATCACCCAGCAGATTGCGCCCAAAAACCTGGACAATCCCTCGAAAAAAGAGCAGGAGCTGATTGCCAAGA
Original	1261	ATAACTCAACAAATAGCACCTAAAAATCTTGATAACCCTAGTAAGAAAGAGCAAGAATTAATAGCCAAAA
Optimized	1331	AGACCGAGAAGGCGAAGTACTTGTCGCTGGAAACCATCAAACTGGCGCTGGAGGAGTTCAACAAGCACCG
Original	1331	AAACTGAAAAAGCAAAATACTTATCTCTAGAAACTATAAAGCTTGCCTTAGAAGAATTTAATAAGCATAG
Optimized	1401	CGACATTGATAAACAATGCCGGTTCGAGGAGATTCTGGCCAATTTTGCCGCCATTCCCATGATCTTCGAT
Original	1401	AGATATAGATAAACAGTGTAGGTTTGAAGAAATACTTGCAAACTTTGCGGCTATTCCGATGATATTTGAT
Optimized	1471	GAGATCGCGCAGAATAAAGACAACCTGGCGCAAATCTCGATCAAATATCAGAATCAGGGCAAAAAAGACC
Original	1471	GAAATAGCTCAAAACAAAGACAATTTGGCACAGATATCTATCAAATATCAAAATCAAGGTAAAAAAGACC
Optimized	1541	TGTTACAAGCCTCGGCCGAAGACGACGTGAAAGCCATCAAGGACCTGTTGGATCAGACGAACAACTTGTT
Original	1541	TACTTCAAGCTAGTGCGGAAGATGATGTTAAAGCTATCAAGGATCTTTTAGATCAAACTAATAATCTCTT
Optimized	1611	GCACAAGCTGAAAATTTTCCACATCAGCCAGAGCGAGGACAAGGCCAACATCTTGGACAAAGACGAGCAC
Original	1611	ACATAAACTAAAAATATTTCATATTAGTCAGTCAGAAGATAAGGCAAATATTTTAGACAAGGATGAGCAT
Optimized	1681	TTCTACTTGGTGTTCGAAGAGTGCTACTTCGAATTGGCCAACATCGTCCCCTGTACAATAAGATTCGCA
Original	1681	TTTTATCTAGTATTTGAGGAGTGCTACTTTGAGCTAGCGAATATAGTGCCTCTTTATAACAAAATTAGAA
Optimized	1751	ATTATATTACGCAGAAGCCCTATAGCGACGAGAAATTCAAGTTGAATTTTGAAAATTCGACCCTGGCGAA
Original	1751	ACTATATAACTCAAAAGCCATATAGTGATGAGAAATTTAAGCTCAATTTTGAGAACTCGACTTTGGCTAA
Optimized	1821	TGGCTGGGACAAGAACAAAGAGCCCGACAACACCGCCATCTTGTTCATCAAGGATGATAAATATTATCTG
Original	1821	TGGTTGGGATAAAAATAAAGAGCCTGACAATACGGCAATTTTATTTATCAAAGATGATAAATATTATCTG
Optimized	1891	GGGGTGATGAACAAAAAGAATAACAAGATCTTTGATGACAAGGCCATCAAGGAGAATAAGGGCGAGGGGT
Original	1891	GGTGTGATGAATAAGAAAAATAACAAAATATTTGATGATAAAGCTATCAAAGAAAATAAAGGCGAGGGTT
Optimized	1961	ACAAGAAAATCGTCTACAAACTGTTGCCCGGCGCCAACAAGATGTTGCCGAAGGTCTTCTTCAGCGCGAA
Original	1961	ATAAAAAAATTGTTTATAAACTTTTACCTGGCGCAAATAAAATGTTACCTAAGGTTTTCTTTTCTGCTAA

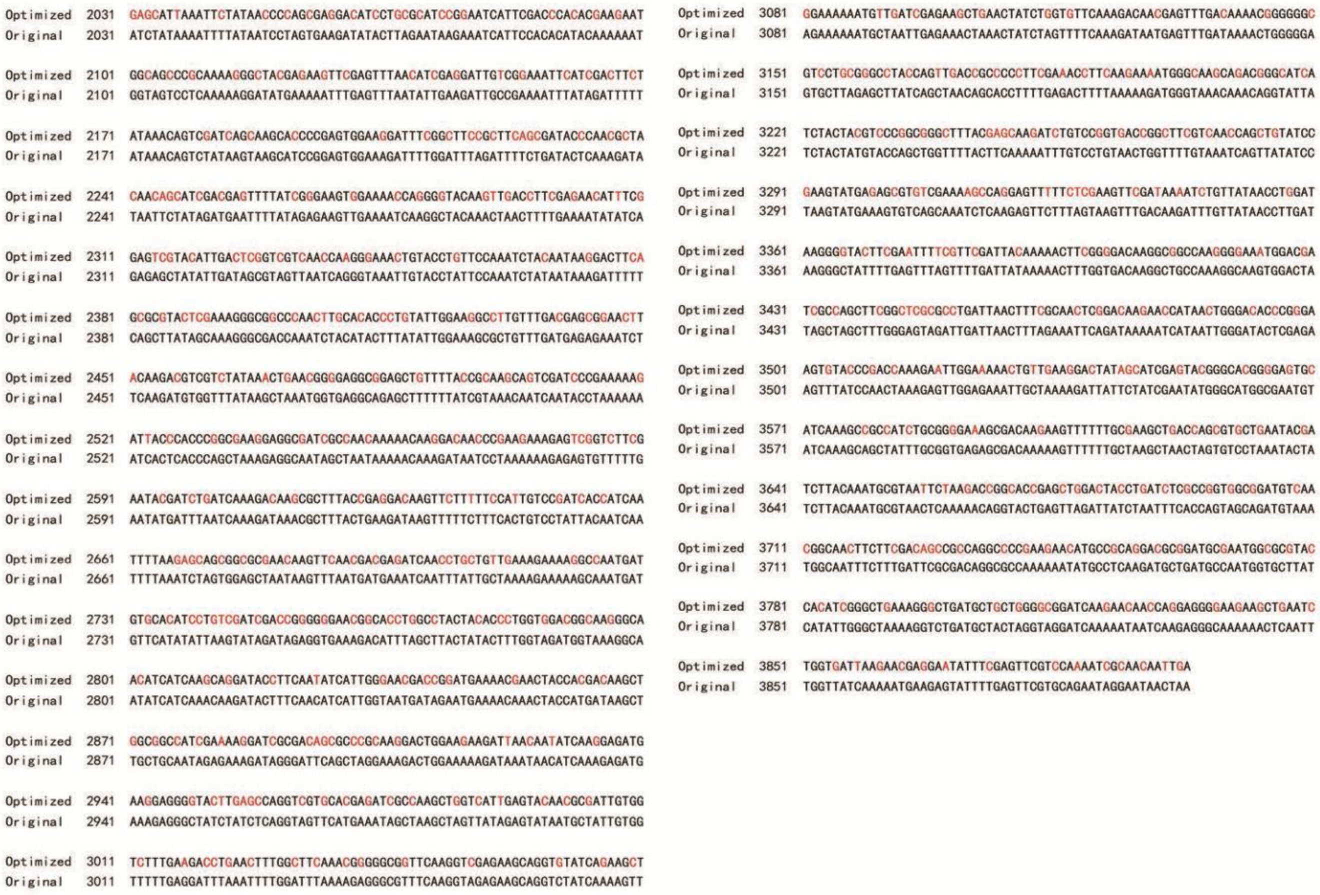

Figure S2. Analysis of FnCpf1 sequence codon-optimized for expression in mycobacteria.

The *FnCpf1* sequence (original) is aligned with the codon-optimized FnCpf1 sequence. Nucleotides replaced in codon-optimized sequences are shown in red.

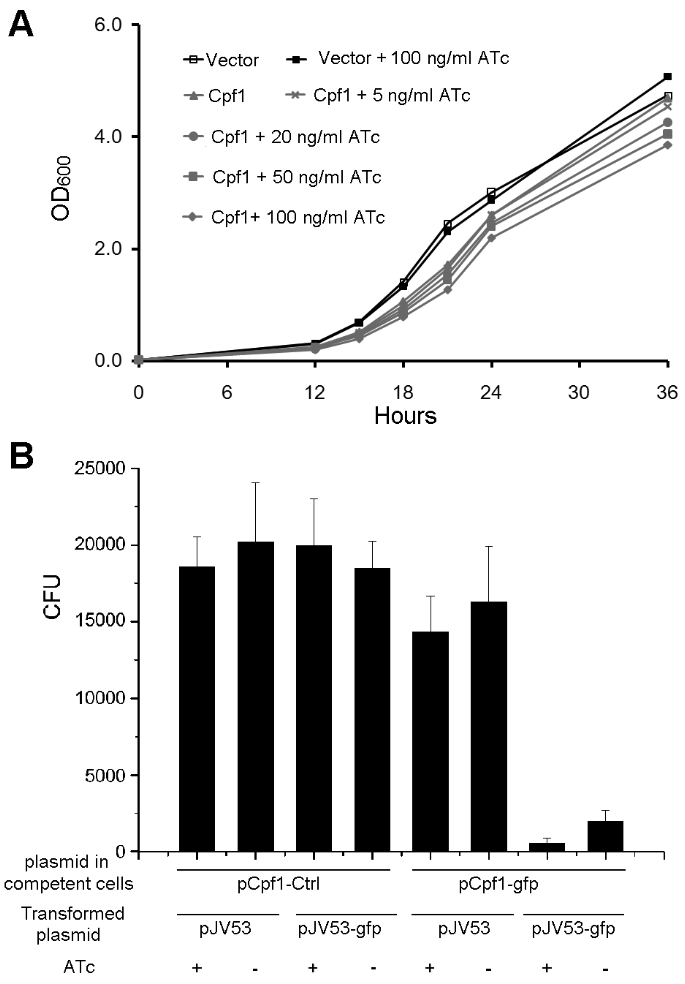

Figure S3. Functional analysis of FnCpf1 in *M. smegmatis*. (A) Influence of FnCpf1 on *in vitro* growth of *M. smegmatis*. FnCpf1 was induced by addition of ATc using plasmid pJV53-Cpf1 in *M. smegmatis*. (B) FnCpf1 expression mediates plasmid interference in *M. smegmatis*. Results are the average of at least two independent experiments, and the error bars depict the standard deviations.

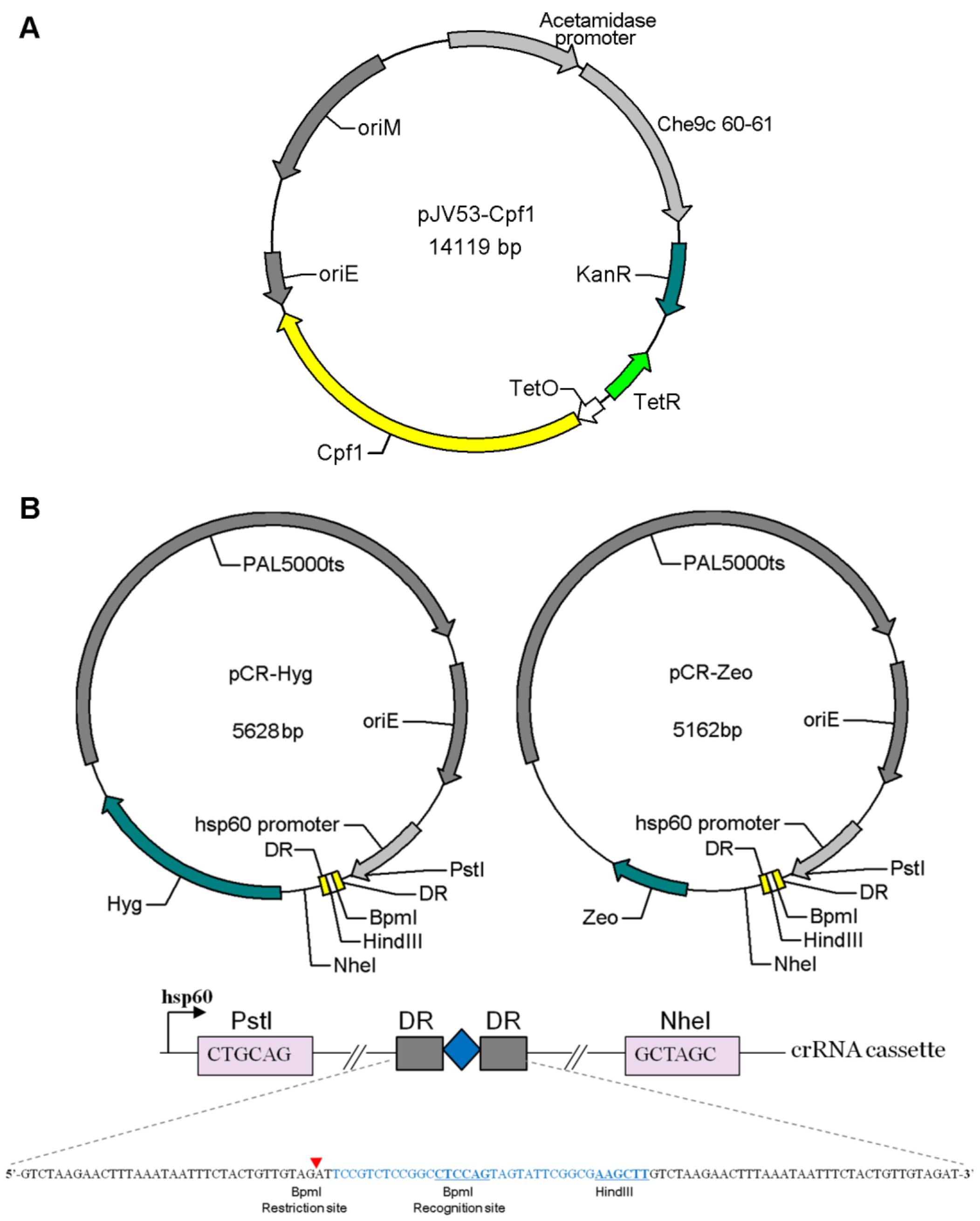

Figure S4. CRISPR-Cpf1-assisted pJV53 based recombineering system. (A) Schematic of pJV53-Cpf1 (GenBank MF193599). (B) Schematic of pCR-Hyg (GenBank MF193598), pCR-Zeo (GenBank MF287366), and the crRNA cassette. The crRNA cassette contains BpmI and HindIII restriction enzyme sites to facilitate cloning of pre-crRNA.

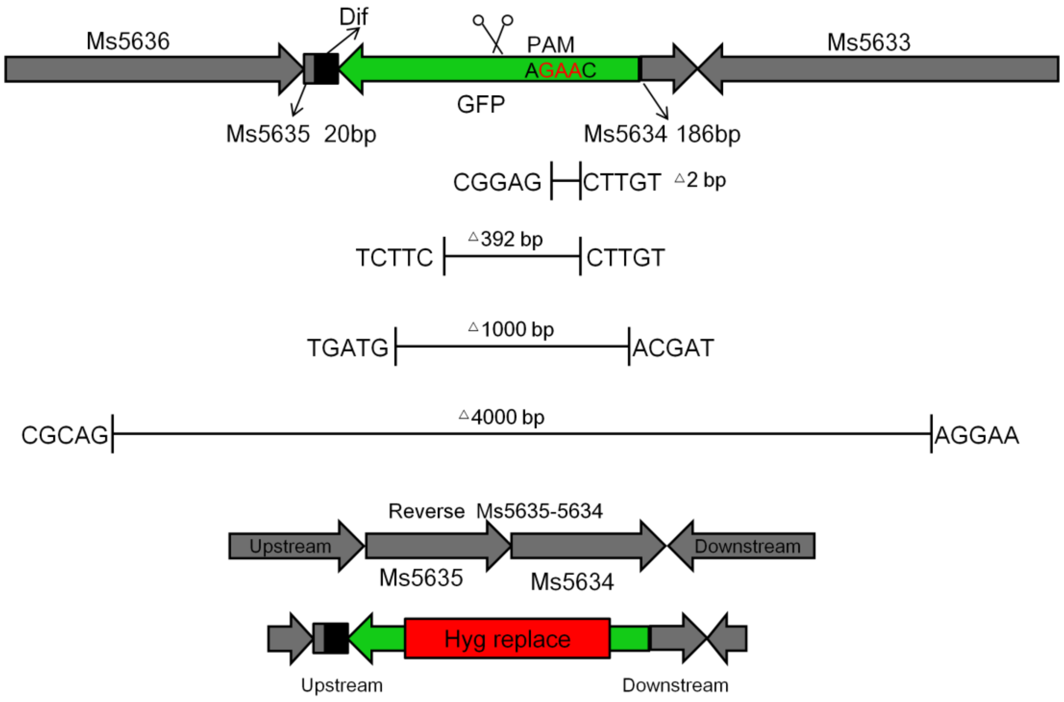

Figure S5. Diagrams of gene deletions and replacements in Figure 6. Deletions of 2-, 392-, 1000-, or 4000-bp were introduced into *M. smegmatis* chromosomal DNA using approximately 1 kb double-stranded DNA fragments. The *gfp* gene was replaced with a dsDNA PCR fragment containing the Hyg-resistance gene or *Ms5635–5634* and its flanking region.

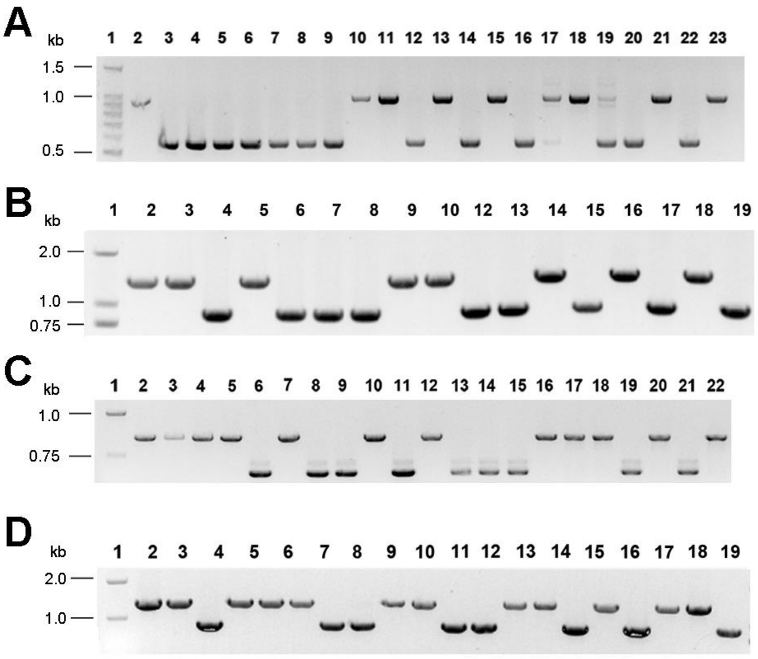

Figure S6. Sequential deletion of four TA genes using CRISPR-Cpf1–assisted recombineering. Colony PCR screening for the deletion of *Ms1277–1288* (A), *Ms1283–1284* (B), *Ms4447–4448* (C), and *Ms5635-5634* (D). Approximately 62% (13/21), 53% (9/17), 45% (9/20), and 47% (8/17) of screened colonies, respectively, were recombinants. Lane 2 in each panel is the wild-type control.

Supplementary Table S1. Plasmids employed in this study

Plasmid	Relevant characteristic(s)	ırce and or referenc
pKD46	*repA*101(ts) *bla araC* P_{araR}-*Red*	Reference(1)
pKD46-Cpf1-Amp	Cpf1 inserted in pKD46 using Gibson cloning	This study
pKD46-Cpf1-Km	kanamycin resistance gene inserted in pKD46-Cpf1-Amp to replace ampicillin resistance gene	This study
pACYC184	*p15Aori cat* Tc^R	Reference(2)
pAC-crRNA-Cm	SacB and synthetic Repeat-AcGFP1-Repeat inserted into pACYC184 using Gibson cloning	This study
pAC-crRNA-Km	kanamycin resistance gene inserted in pAC-crRNA-Cm to replace chloramphenicol resistance gene	This study
pAC-crRNA-Str	streptomycin resistance gene inserted in pAC-crRNA-Cm to replace chloramphenicol resistance gene	This study
pcrRNA-ctrl	non-target protospacer inserted in pAc-crRNA-Cm digested with *Bpm*I	This study
pcrRNA-lacZ	Protospacer of *lacZ* in pAc-crRNA-Cm	This study
pcrRNA-aroA	Protospacer of *aroA* in pAc-crRNA-Cm	This study
pcrRNA-hmsT	Protospacer of *hmsT* in pAc-crRNA-Cm	This study
pcrRNA-y4098	Protospacer of *y4098* in pAc-crRNA-Cm	This study
pcrRNA-caf1R1	Protospacer of *caf1R* R129A in pAc-crRNA-Cm	This study
pcrRNA- caf1R2	Protospacer of *caf1R* R146A in pAc-crRNA-Cm	This study
pcrRNA- caf1R3	Protospacer of *caf1R* R191A in pAc-crRNA-Cm	This study

pcrRNA- caf1R4	Protospacer of *caf1R* R245A in pAc-crRNA-Cm	This study
pMV261	Shuttle vector; replicates extrachomosomally in both *E.coli* and mycobacterium.(oriM; oriE; Kn^r)	Reference(3)
pJV53	Shuttle vector containing Che9c genes 60-61 under control of acetamidase promoter; replicates extrachromosomally in both *E.coli* and mycobacterium.(oriM; oriE; Kn^r; Che9c)	Reference(4)
pJV53-GFP	GFP cassette inserted in pJV53.(oriM; oriE; Kn^r; Che9c; Gfp)	Reference(5)
pMV261-Cpf1	Optimized Cpf1 under control of the P_{myc1}*tetO* promoter inserted in pMV261.(oriM; oriE; Kn^r; Cpf1)	This study
pJV53-Cpf1	Optimized Cpf1 under control of the P_{myc1}*tetO* promoter inserted in pJV53.(oriM; oriE; Kn^r; Che9c; Cpf1)	This study
pcrRNA-ctrl	Shuttle vector containing a direct repeats-pre crRNA-direct repeats cassette downstream to Hsp60 promoter; replicates extrachromosomally in both *E.coli* and mycobacterium.(pBR322 ori; pAL5000ts; crRNA cassette; Hyg^r)	This study
pcrRNA-gfp1	Shuttle vector containing a direct repeats-gfp1 pre crRNA-direct repeats cassette downstream to Hsp60 promoter; replicates extrachomosomally in both *E.coli* and mycobacterium.(pBR322 ori; pAL5000ts; *gfp*1 crRNA cassette; Hyg^r)	This study
pCpf1-ctrl	Optimized Cpf1 under control of the P_{myc1}*tetO* promoter inserted in pcrRNA-ctrl. (pBR322 ori; pAL5000ts; Cpf1; crRNA cassette; Hyg^r)	This study
pCpf1-gfp	Optimized Cpf1 under control of the P_{myc1}*tetO* promoter inserted in pcrRNA-gfp1. (pBR322 ori; pAL5000ts; Cpf1; *gfp* crRNA cassette; Hyg^r)	This study
pCR-Hyg	Shuttle vector containing crRNA cassette which inserted *Bpm*I and *Hin*dIII sites between Direct	This study

	repeats downstream to Hsp60 promoter; replicates extrachomosomally in both *E.coli* and mycobacterium. (pBR322 ori; pAL5000ts; Hyg^r)	
pCR-Zeo	Zeo replace hyg resistance in pCR-Hyg. (pBR322 ori; pAL5000ts; Zeo^r)	This study
pCR-Hyg-gfp	gfp crRNA inserted in pCR-Hyg digested with *Bpm*I and *Hin*dIII. (pBR322 ori; pAL5000ts;gfp crRNA cassette; Hyg^r)	This study
pCR-Zeo-gfp	gfp crRNA inserted in pCR-Zeo digested with *Bpm*I and *Hin*dIII. (pBR322 ori; pAL5000ts;gfp crRNA cassette; Zeo^r)	This study
pYC847	the Ms5635-5634::*gfp* cassette with flanking regions inserted in pUC19.(pBR322 ori; Ap^r)	This study
pYC848	pUC19 containing dsDNA homologous arms for the 4000bp deletion in the Ms5635-5634::*gfp*.(pBR322 ori; Ap^r; dsDNA homologous arms)	This study
pYC710	Ms1283-1284 homologous arms inserted into pUC-Hyg.(pBR322 ori; Ap^r; Ms1283-1284 homologous arms)	Reference5
pYC711	Ms1277-1278 homologous arms inserted into pUC-Hyg.(pBR322 ori; Ap^r; Ms1277-1278 homologous arms)	Reference 5
pYC738	Ms4447-4448 homologous arms inserted into pUC-Hyg.(pBR322 ori; Ap^r; Ms4447-4448 homologous arms)	Reference 5
pYC799	Ms5635-5634 homologous arms inserted into pUC-Hyg.(pBR322 ori; Ap^r; Ms5635-5634homologous arms)	Reference 5
pYC984	pUC57 containing Ms1277-1278 homologous arms without dif-hyg-dif cassette.(pBR322 ori; Ap^r; Ms1277-1278 homologous arms)	This study
pYC985	pUC57 containing Ms1283-1284 homologous arms without dif-hyg-dif cassette.(pBR322 ori; Ap^r;	This study

	Ms1283-1284 homologous arms)	
pYC986	pUC57 containing Ms4447-4448 homologous arms without dif-hyg-dif cassette.(pBR322 ori; Ap^r; Ms4447-4448 homologous arms)	This study
pYC987	pUC57 containing Ms5635-5634 homologous arms without dif-hyg-dif cassette.(pBR322 ori; Ap^r; Ms5635-5634 homologous arms)	This study
pYC1009	Ms1277-1278 crRNA inserted in pCR-Hyg digested with *Bpm*I and *Hin*dIII. (pBR322 ori; pAL5000ts; Ms1277-1278 crRNA cassette; Hyg^r)	This study
pYC1010	Ms1283-1284 crRNA inserted in pCR-Zeo digested with *Bpm*I and *Hin*dIII. (pBR322 ori; pAL5000ts; Ms1283-1284 crRNA cassette; Zeo^r)	This study
pYC983	Ms4447-4448 crRNA inserted in pCR-Hyg digested with *Bpm*I and *Hin*dIII. (pBR322 ori; pAL5000ts; Ms4447-4448 crRNA cassette; Hyg^r)	This study
pYC1011	Ms5635-5634 crRNA inserted in pCR-Zeo digested with *Bpm*I and *Hin*dIII. (pBR322 ori; pAL5000ts; Ms5635-5634 crRNA cassette; Zeo^r)	This study

Supplementary Table S2. Oligonucleotides used in this study

Primer name	Sequence 5′–3′
crRNA-lacZ top	tccgaccgcacgccgcatccagcgctgt
crRNA-lacZ bottom	agcgctggatgcggcgtgcggtcggatc
lacZ oligo for leading	tcagcgctggatgcggcgtgcggtcggcttagaccagaccgttcatacagaactggcga
lacZ oligo for lagging	tcgccagttctgtatgaacggtctggtctaagccgaccgcacgccgcatccagcgctga
lacZ oligo for deletion	ctttaatgatgatttcagccgcgctgtactggaggctgaaaagccgggcacatcagcgcctg gcagcagtggcgtctgg
aroA crRNA top	tctcaccgcactgttaatgactgcgcgt
aroA crRNA bottom	gcgcagtcattaacagtgcggtgagatc
aroA oligo for deletion	taaagaaagatttggctatttattgcccgttgttcattcacatgaactcaactctctacaacagaa ataaaaaccccac
caf1R R129A crRNA top	taaccacgaatctgttaccttaaagagt
caf1R R129A crRNA bottom	tctttaaggtaacagattcgtggttatc
caf1R R129A oligo for leading	ggaaattaactgtgaataccttoaaccagctatctgttaccttaaagagagaaatataa
caf1R R129A oligo for lagging	ttatatttctctctttaaggtaacagatagctggttgaaggtattcacagttaatttcc
caf1R R146A crRNA top	tattttagggatttagtgttctactcgt
caf1R R146A crRNA bottom	gagtagaacactaaatccctaaaatatc
caf1R R146A oligo for leading	aaatataattggtcaatgctttaattttgctgatttagtgttctactctgggatagatt
caf1R R146A oligo for lagging	aatctatcccagagtagaacactaaatcagcaaaattaaagcattgaccaattatattt
caf1R R191A crRNA top	tcaagaacggttgtttgggataggaagt
caf1R R191A crRNA bottom	ttcctatcccaaacaaccgttcttgatc
caf1R R191A oligo for leading	tcatgataaaacgaatgacattattgcagctacggttgtttgggataggaataagcatt
caf1R R191A oligo for lagging	aatgcttattcctatcccaaacaaccgtagctgcaataatgtcattcgttttatcatga
caf1R R245A crRNA top	taataagcgggatggttacgatgtgggt
caf1R R245A crRNA bottom	ccacatcgtaaccatcccgcttattatc
caf1R R245A oligo for leading	ctctttgcctatttataatttaaataaggctgatggttacgatgtggaggtcataaaaa
caf1R R245A oligo for lagging	tttttatgacctccacatcgtaaccatcagccttatttaaattataaataggcaaagag
hmsT mutation crRNA top	tactcactgaacatacggacgctctagt
hmsT mutation crRNA bottom	tagagcgtccgtatgttcagtgagtatc
hmsT mutation oligo for lagging	atgtacagtcttccccttgattaacagctcgaacatacgtaggctctatgccagttattatttttaa
y4098 mutation crRNA top	tgggcatacaaactttattttgactcgt
y4098 mutation crRNA bottom	gagtcaaaataaagtttgtatgcccatc
y4098 mutationoligo for lagging	ttctaaagctgataattagggcataggagctttattttatgtcgtacgggaaagaaagcaaacttg
gfp crRNA1 oligo for leading	tggcgtcgccctcaccctcgccggagacttattacttgtggccgttgacgtcaccgtcca
gfp crRNA1 oligo for lagging	tggacggtgacgtcaacggccacaagtaataagtctccggcgagggtgagggcgacgcca
gfp crRNA2 oligo for leading	cgtggcgcttcatgtggtccgggtagcgctactagcactggacgccgtaggtcagggtgg
gfp crRNA2 oligo for lagging	ccaccctgacctacggcgtccagtgctagtagcgctacccggaccacatgaagcgccacg
gfp crRNA1 point mutation1	gctggacggtgacgtcaacggccacaagtaatccgtctccggcgagggtgagggcgacg
gfp crRNA1 point mutation2	gctggacggtgacgtcaacggccacaagtagtccgtctccggcgagggtgagggcgacg
gfp crRNA1 point mutation3	gctggacggtgacgtcaacggccacaagtgatccgtctccggcgagggtgagggcgacg
gfp crRNA1 point mutation4	tggacggtgacgtcaacggccacaagttctaagtctccggcgagggtgagggcgacgcca
gfp crRNA1 point mutation5	tggacggtgacgtcaacggccacaagttctcctaatccggcgagggtgagggcgacgcca

gfp crRNA1 point mutation6	tgacgtcaacggccacaagttctccgtctaaggcgagggtgagggcgacgccacctacg
gfp crRNA1 point mutation7	cgtcaacggccacaagttctccgtctcctgagagggtgagggcgacgccacctacggca
gfp crRNA1 point mutation8	caacggccacaagttctccgtctccggctagggtgagggcgacgccacctacggcaagc
gfp crRNA1 point mutation9	caacggccacaagttctccgtctccggctaaggtgagggcgacgccacctacggcaagc
gfp crRNA1 point mutation10	cggccacaagttctccgtctccggcgagtgagagggcgacgccacctacggcaagctga
gfp crRNA1 point mutation11	ccacaagttctccgtctccggcgagggttagggcgacgccacctacggcaagctgaccc
gfp crRNA1 point mutation12	caagttctccgtctccggcgagggtgagtaagacgccacctacggcaagctgaccctga
gfp crRNA1 point mutation13	ttctccgtctccggcgagggtgagggctagtaaacctacggcaagctgaccctgaagttc
gfp crRNA1 insertion1	ggacggtgacgtcaacggccacaagttctgccgtctccggcgagggtgagggcgacgcc
gfp crRNA1 insertion2	gacggtgacgtcaacggccacaagttctcacgtctccggcgagggtgagggcgacgcca
gfp crRNA1 insertion3	cggtgacgtcaacggccacaagttctccgatctccggcgagggtgagggcgacgccacc
gfp crRNA1 insertion4	acgtcaacggccacaagttctccgtctcctggcgagggtgagggcgacgccacctacgg
gfp crRNA1 insertion5	tcaacggccacaagttctccgtctccggctgagggtgagggcgacgccacctacggcaa
gfp crRNA1 insertion6	acggccacaagttctccgtctccggcgagcggtgagggcgacgccacctacggcaagct
gfp crRNA1 insertion7	ggccacaagttctccgtctccggcgagggctgagggcgacgccacctacggcaagctga
gfp crRNA1 insertion8	gccacaagttctccgtctccggcgagggttgagggcgacgccacctacggcaagctgac
gfp crRNA1 insertion9	acaagttctccgtctccggcgagggtgagaggcgacgccacctacggcaagctgaccct
gfp crRNA1 delete1	ggacggtgacgtcaacggccacaagttctcgtctccggcgagggtgagggcgacgccac
gfp crRNA1 delete2	cggtgacgtcaacggccacaagttctccgctccggcgagggtgagggcgacgccaccta
gfp crRNA1 delete3	ggtgacgtcaacggccacaagttctccgtcccggcgagggtgagggcgacgccacctac
gfp crRNA1 delete4	gacgtcaacggccacaagttctccgtctccgcgagggtgagggcgacgccacctacggc
gfp crRNA1 delete5	tcaacggccacaagttctccgtctccggcggggtgagggcgacgccacctacggcaagc
gfp crRNA1 delete6	acggccacaagttctccgtctccggcgaggtgagggcgacgccacctacggcaagctga
oligo delete 5bp	agctggacggtgacgtcaacggccacaagtgtctccggcgagggtgagggcgacgccac
oligo delete 10bp	ctggacggtgacgtcaacggccacaagttcgcgagggtgagggcgacgccacctacggc
oligo delete 20bp	ctggacggtgacgtcaacggccacaagttcgggcgacgccacctacggcaagctgaccc
oligo delete 418bp	ttgtcctgcgtgtgctcggtcgagtaggctctgggagtaaagacgcgtgccgaggtcaagttc gagggcgacaccctgg
oligo delete1000bp	taaatctacttgtacagctcgtccatgccgtgggtgatgacgatccaatatttcactagtctgatc ggcaagatcaagc
oligo insert 5bp	gacggtgacgtcaacggccacaagttctaatgtccgtctccggcgagggtgagggcgac
oligo insert 10bp	cggtgacgtcaacggccacaagttctaatagtgaatccgtctccggcgagggtgagggc
Ms1521 T49V crRNA top	attgcgcatgttcttgtcgatgcccgta
Ms1521 T49V crRNA bottom	agcttacgggcatcgacaagaacatgcgcaatct
*Ms1521*T49V oligo for lagging	gcagaacggtcacctgatcgtcgaccaggtccttagtgcgcatgttcttgtcgatgccc
Ms1521 C86S crRNA top	atctaccagggcctgcgccaccgccgta
Ms1521 C86S crRNA bottom	agcttacggcggtggcgcaggccctggtagatct
*Ms1521*C86S oligo for lagging	ggccacggcggtggcgcaggccctggtaactaccgatctcgatcttgcggcggatgtcg
Ms1283-1284 crRNA top	atggcgagccaggtggtcgcgaactcga
Ms1283-1284 crRNA bottom	agcttcgagttcgcgaccacctggctcgccatct
*Ms4447-4448*crRNA top	atgaatcgagccaacgccagccaccgga
Ms4447-4448 crRNA bottom	agcttccggtggctggcgttggctcgattcatct
*Ms5635-5634*crRNA top	atcgtctccgtcgaagttccattgccga
Ms5635-5634 crRNA bottom	agcctcggcaatggaacttcgacggagacgatct

oligo delete Ms1277-1278	tactattgtgcgtatgccgtcgctgaacatcgactcggaggacctcatgaacgaggtggcca cccgtgac
FnCpf1 insert to pKD46 F	gattttccagtctgattgatgcaatatttgtcttag
FnCpf1 insert to pKD46 R	cgaagtggcacaagtaagcccttatctttatg
pKD46F	gcttacttgtgccacttcggattatcccgtgac
pKD46R	gcatcaatcagactggaaaatcagagggcagg
SacB F	acttttggctcgagcattcaaatatgtatccgctc
SacB R	cgttaaatagccgcttatcatatgcacagatgaaaacgg
pACYC184-F	atctgtgcatatgataagcggctatttaacgac
pACYC184-R	gataaactaccgcattaaagcttatcgatgataagctg
Cpf1insert to pJV53 F	ggactagtggccatgatggcataaaacg
Cpf1insert to pJV53 R	caaatactagtaggactctgcag
pMV261-Cpf1F	ataagaatgcggccgcgcccatggtcattaagaccc
pMV261-Cpf1R	cggggtaccacgaagttattcaattgttgcg
pSL001F	atgccgatatcctattggca
pSL001R	tacgttgcgtcgagagacga
pSL003 insert crRNA F	tcgtctctcgacgcaacgtatgttaggtggcggtacttgg
pSL003 insert crRNA R	tgccaataggatatcggcattctagacggtgaccacaacg
del*MS5365-5634+gfp*F1	ggggtaccgtgtggagatagctggtcga
del*MS5365-5634+gfp*F2	ggggtaccttcgtgtcctatctcgtggc
del*MS5365-5634+gf*pR	cccaagctttactggaacaaccctgaggc
gfp del2bpF	cttgtggccgttgacgtcaccgtccagctcgaccaggatc
gfp del2bpR	gtgacgtcaacggccacaagctccgtctccggcgagggt
gfp del392bpF	tggccttgatgccgttcttcacttcaagacccgccacaacat
gfp del392bpR	gaagaacggcatcaaggccagtgaacagctcctcgccctt
gfp del1000bpF	acgatccaatatttcactagtctgatcggcaagatcaagccca
gfp del1000bpR	gactagtgaaatattggatcgtcatcacccacggcatggacg
Ms5636 homologous arms F	ggggtacctgttcgtcatcgtgctgtcg
Ms5636 homologous arms R	gctctagactgcgtgtagatggtcagca
Ms5633 homologous arms F	gctctagaaggaactcgtcgggttcgaa
Ms5633 homologous arms R	acatgcatgcgtacacgtggacacaactg
hyg replace F	ctagctagcgtccgctgtgacacaagaat
hyg replace R	acatgcatgctatgggtctagatcaggcgc
*Ms1277-1278*F	ggatccactagtcggaggacctcatgaacgagg
*Ms1277-1278*R	actagtggatcccgtgcgcgaacttcttcagc
*Ms1283-1284*F	acggatccctagttcgtgcaggcctgaattacggcgact
*Ms1283-1284*R	gcacgaactagggatccgtcggcttccgggtgtttgatg
*Ms4447-4448*F	cgaaacagacatggtcaggaccgaccg
*Ms4447-4448*R	tgtttcgggtgcattcacgtcgggga
*Ms5635-5634*F	ctggacaaggcgatcggcaagatcaagcccaccgacaacg
*Ms5635-5634*R	ttgccgatcgccttgtccaggaatcccatgggctcagcct
P1(*Ms1277-1278*)	tctggcaatggcatccaaca
P2(*Ms1277-1278*)	tcgcgtcgtaatccacgatc
P3(*Ms4447-4448*)	cgaatacaccacgatgcccc

P4(*Ms4447-4448*)	tcatggatcgtgccgaagca
P5(*Ms1283-1284*)	gaactcgggctacggtccaa
P6(*Ms1283-1284*)	aactgctcgacagcttcccg
P7(*Ms5635-5634*)	accaagaccgtgtactcggt
P8(*Ms5635-5634*)	cagttacggcaaggtgctca

References

1. **Datsenko KA, Wanner BL.** 2000. One-step inactivation of chromosomal genes in Escherichia coli K-12 using PCR products. Proc Natl Acad Sci U S A **97:**6640-6645.
2. **Chang ACYaC, S.N.** 1978. Construction and characterization of amplifiable multicopy DNA cloning vehicles derived from the P15A cryptic miniplasmid. J Bacteriol**:**1141-1156.
3. **Stover CK, de la Cruz VF, Fuerst TR, Burlein JE, Benson LA, Bennett LT, Bansal GP, Young JF, Lee MH, Hatfull GF, Snapper SB, Barletta RG, Jacobs WR, Bloom BR.** 1991. New use of BCG for recombinant vaccines. Nature **351:**456-460.
4. **van Kessel JC, Hatfull GF.** 2007. Recombineering in Mycobacterium tuberculosis. Nat Methods **4:**147-152.
5. **Mao XJ, Yan MY, Zhu H, Guo XP, Sun YC.** 2016. Efficient and simple generation of multiple unmarked gene deletions in Mycobacterium smegmatis. Sci Rep **6:**22922.
